# Supplementary material for: From sequence to enzyme mechanism using multi-label machine learning
Source: BMC Bioinformatics. 2014 May 19;15:150. doi: 10.1186/1471-2105-15-150 (PMC4229970; doi:10.1186/1471-2105-15-150)
Supplement: Additional file 2 — Java code of ml2db. Additional file ml2db_code.tar.gz contains the Java source code to run the multi-label machine learning experiments and save the results to database. The code’s Javadoc is included. [file 1471-2105-15-150-S2.zip › additional file 2/ml2db/utils/doc/uk/ac/standrews/utils/main/database/package-summary.html]

uk.ac.standrews.utils.main.database


JavaScript is disabled on your browser.


- Overview
- Package
- Class
- Use
- Tree
- Deprecated
- Index
- Help

- Prev Package
- Next Package

- Frames
- No Frames

- All Classes

# Package uk.ac.standrews.utils.main.database

- Class Summary

  | Class | Description |
  | --- | --- |
  |  |  |
  | --- | --- |
  | DbConn | A DbConnectionProperties object contains the properties needed to connect to a database through JDBC. |
  | DbConnectionSingleton | A singleton database connection |
  | DbCreator | Class |
  | DbManaged | Managed is a class which has a `DbManager` (an object that coordinates its database activities) |
  | DbManager | A database manager provides object to create a database, write and read from it. |
  | DbManagerSingleton | Class |
  | DbReader | Class to read from a database. |
  | DbUtils | Database utilities (to create sql code etc.) |
  | DbWriter | Provides methods to write to a database. |
  | Managed | A class with a manager object |
  | Manager | A manager manages: for example, for a database object, the `DbManager` manages a `DbCreator` to create the tables, `DbReader` to read data from and `DbWriter` to write data to the database. |
  | ResultSetUtils | Class |
  | SqlUtils | Class |
  | Table | An SQL database table definition (table name + columns) |
  | TableColumn | A column object contains all the information to create, fill and delete a database table column |
  | TableCreator | Class to create a table object in the database. |
  | TableManager | Class to create/read/write to a database table. |
  | TableReader | Class |
  | TableRow | Contains the data for a generic row (a list of [table column, value] entries) to be written to a database table. |
  | TableWriter | A TableWriter object contains all the information to create, fill and delete a database table. |

- Overview
- Package
- Class
- Use
- Tree
- Deprecated
- Index
- Help

- Prev Package
- Next Package

- Frames
- No Frames

- All Classes
